# *Momordica cochinchinensis* (Gấc) Seed Extracts Induce Apoptosis and Necrosis in Melanoma Cells

**DOI:** 10.3390/ph16010100

**Published:** 2023-01-09

**Authors:** Dao Nguyen, Jessica Holien, Chaitali Dekiwadia, Thilini Thrimawithana, Terrence Piva, Tien Huynh

**Affiliations:** 1 School of Science, RMIT University, P.O. Box 71, Bundoora 3083, Australia; 2 Faculty of Agriculture and Forestry, Tay Nguyen University, 567 Le Duan Street, Buon Ma Thuot City 63000, Vietnam; 3 RMIT Microscopy and Microanalysis Facility, GPO 2476, Melbourne 3001, Australia; 4 School of Health and Biomedical Sciences, RMIT University, P.O. Box 71, Bundoora 3083, Australia

**Keywords:** *M. cochinchinensis*, gac fruit, melanoma, MAPK signalling pathway, NF-kB, Nrf2, TNFR1, BRAF oncogene, cancer

## Abstract

*Momordica cochinchinensis* is a herbal medicine used throughout Asia and this study investigated the antimelanoma potentials and molecular mechanisms of *M. cochinchinensis* seed with emphasis on extraction to optimise bioactivity. Overall, the aqueous extract was superior, with a wider diversity and higher concentration of proteins and peptides that was more cytotoxic to the melanoma cells than other extraction solvents. The IC50 of the aqueous extract on melanoma cells were similar to treatment with current anticancer drugs, vemurafenib and cisplatin. This cytotoxicity was cancer-specific with lower cytotoxic effects on HaCaT epidermal keratinocytes. Cytotoxicity correlated with MAPK signalling pathways leading to apoptosis and necrosis induced by triggering tumour necrosis factor receptor-1 (TNFR1), reducing the expression of nuclear factor kappa B (NF-kB), and suppression of BRAF/MEK. This efficacy of *M. cochinchinensis* seed extracts on melanoma cells provides a platform for future clinical trials as potent adjunctive therapy for metastatic melanoma.

## 1. Introduction

Cancer originates from mutations to the cell’s DNA resulting in uncontrolled growth. It is one of the main causes of death in the general population, and in 2020 it was estimated to have caused 9.5 million deaths worldwide with the number of new cases at ~19.3 million [1]. Melanoma is the deadliest form of skin cancer due to its high metastatic rate [1]. Although accounting for ~4% of skin cancer cases, it is responsible for ~80% of skin cancer-related deaths [2]. It was estimated that there were ~300,000 new melanoma cases worldwide in 2020 [1]. Currently the treatment options for metastatic melanoma are limited, with the median survival time of patients at ~8–9 months, while the 3-year overall survival rate is <15% [2,3]. A malfunctioning apoptotic mechanism is the main reason for the poor response of melanomas to conventional anti-cancer therapies [4]. BRAF mutations occur in ~50% of melanomas, the majority of which are BRAF*^V600E^* [2]. BRAF mutations drive constitutive activation of BRAF-MEK-ERK-MAPK signalling pathway (MAPK), which promotes melanoma cell proliferation and survival [2,3,5]. Thus, targeting MAPK pathway intermediates is a promising therapy for the treatment of BRAF mutated metastatic melanomas. Several BRAF and MEK inhibitors have been approved for treatment of these melanomas, such as vemurafenib, dabrafenib, encorafernib, trametinib, binimetinib, selumetinib and cobimetinib [3,5]. However, the main challenge of treatment with these inhibitors is acquired resistance, which frequently occurs after 6–8 months of treatment [6,7]. The acquired resistance of BRAF/MEK inhibitors is often associated with activation of nuclear factor erythroid 2-related factor 2 (Nrf2) [8]. Nrf2 activates antioxidant enzymes enhancing the cell’s antioxidant defence against oxidative stress induced by these BRAF/MEK inhibitor(s) [8]. Overexpression of Nrf2 also enhances epidermal growth factor receptors, which subsequently promotes melanoma migration and angiogenesis [9]. Therefore, anticancer agents targeting the Nrf2 antioxidant pathway could be used to prevent the emergence of resistance of these melanomas to anti-MAPK therapy.

Furthermore, the activation of other signalling pathways such as the tumour necrosis factor alpha (TNF-α)/nuclear factor kappa B (NF-kB) pathway enhances melanoma cell growth, and contributes to the failure of chemotherapy treatment including BRAF inhibitors [10]. TNF-α binds to two receptors on the cell surface: TNFR1 and TNFR2. The NF-kB pathway induces cell proliferation and can be triggered when TNF-α binds to TNFR2; however, when this ligand binds to TNFR1, it can enhance apoptosis in melanoma cells [4,11].

Phytochemicals are important sources for discovering anticancer agents. Various anticancer drugs have been developed from plant products such as vincristine, camptothecin, vinblastine, topotecan, taxol, irinotecan and podophyllotoxin [12]. *Momordica cochinchinensis* (Lour.) Spreng is botanically classified in the Cucurbitaceae family, and it is geographically restricted to tropical climates predominantly in Southeast Asia [13]. The main nutritional and medicinal usages of this plant is its fruit. The fruit is comprised of black seeds, which are covered by an oily red membrane (aril), an orange spongy mesocarp (pulp) and an orange-red peel. The aril is commercially extracted for its high levels of lycopene and β-carotenoid [14]. The seed accounts for ~16% of the fruit, and has been used in traditional Asian medicine to treat ulcers and inflammation [15,16]. It is reported that extracts of *M. cochinchinensis* seeds possess cytotoxic activity against cancers such as liver HepG [17], colon 20–26 [17], breast ZR-75-30 [18], breast MDA-MB-301 [19], gastric MKN-28 and SGC7901 [20], lung carcinoma A549 [21] and oesophageal squamous Kyse30 carcinoma cells [22].

Gac seed extracts are reported to be cytotoxic to B16 murine tumour cells [23], MM96L and MM418-C1 human melanoma cells (BRAF*^V600E^* oncogene) [24,25]. However, the molecular mechanism(s) underlying the activity of these extracts, in particular on the MAPK pathway intermediates has not been investigated.

Furthermore, various proteins and peptides from *M. cochinchinensis* seed extracts have been reported to exert anticancer activities such as unknown 35 kDa [17] and 30 kDa proteins [26] as well as cochinin B [27], and MCoCC-1 and MCoCC-2 peptides [25]. However, when these compounds were isolated, they exerted a reduced cytotoxic effect than did the equivalent concentration of crude extracts [23,25]. This suggests that the seed contains multiple potent anticancer compounds which may display synergistic effects in its native matrix. Therefore, this study aimed to (1) determine the best extraction solvent for optimal antimelanoma activity compared to current anticancer drugs, (2) investigate the effect of the extracts on the expression of genes involved in BRAF/MEK and TNFR1/NF-kB induced cell death pathways in melanoma cells, and (3) to evaluate responsible proteins and peptides in these extracts. Results from this study will provide the basis for further research on the antimelanoma activity of *M. cochinchinensis* seeds and assess its potential as a novel herbal-inspired source of anticancer compounds.

## 2. Results

### 2.1. Viability and Toxicity on Cancer and Control Cells

**Figure 1:**
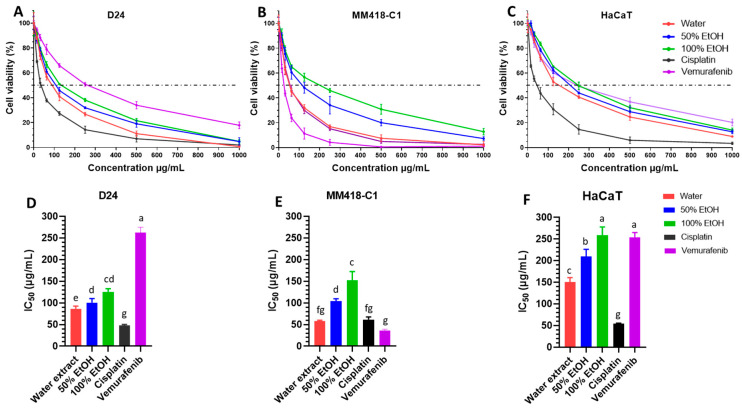
Effect of *M. cochinchinensis* seed extracts on the cell viability and half inhibition concentration (IC50) of D24 and MM418-C1 melanoma and HaCaT cells (*n* = 3). Dose response of cells (% viability) after 48 h treatment with different concentrations of water, 50% EtOH and 100% EtOH extracts, cisplatin and vemurafenib on D24 melanoma cells (**A**), MM418-C1 melanoma cells (**B**), and HaCaT cells (**C**). Cell viability (%) was expressed as the average ratio of the treated divided by the untreated control (the dotted lines represented 50% cell viability); half inhibition concentration (IC50) of the seed extracts for D24 melanoma cells (**D**), MM418-C1 melanoma cells (**E**), and HaCaT cells (**F**). The results were presented as the mean ± SEM, different letters indicated statistical significance at *p* ≤ 0.05 between different treatments (Analysis by One-way ANOVA with Turkey’s LSD test).

The viability of all three cell lines (MM418-C1, D24 and HaCaT cells) treated with *M. cochinchinensis* seed extracts or control drugs at different concentrations (0, 15.625, 31.25, 65.5, 125, 250, 500 and 1000 µg/mL) for 48 h, was measured using the CCK-8 assay. The seed extracts decreased the viability of all the cell lines in a dose dependent manner (Figure 1A–C). The materials extracted in the different solvents had an impact on the viability of these cells. The water extracts were the most potent indicated by the low IC50 values on D24 (86 μg/mL) and MM418-C1 cells (58 μg/mL), followed by the 50% and 100% ethanol extracts (Figure 1D,E).

MM418-C1 melanoma cells containing the BRAF*^V600E^* oncogene as well as D24 melanoma cells containing BRAF*^WT^* were used to test for specific cytotoxic effects of *M. cochinchinensis* extracts on BRAF mutation, while human immortalised keratinocytes (HaCaT) was used to compare the cytotoxicity of the extracts on a non cancerous epithelial cell. Cisplatin was cytotoxic against all cell lines as indicated by the low IC50 values (Figure 1). IC50 values for cisplatin on the D24 and MM418-C1 melanoma cells were 48.3 and 61.7 µg/mL, respectively (Figure 1D,E). The cytotoxicity of the water extract was less than cisplatin in D24 (by 38.3 µg/mL) (Figure 1D) but was comparable to that seen in the MM418-C1 melanoma cells (Figure 1E). The anti-BRAF*^V600E^* drug, vemurafenib [28] was as expected and more cytotoxic to BRAF*^V600E^* MM418-C1 melanoma cells than to D24 cells (Figure 1A–C). The water extract was 3 times more cytotoxic than vemurafenib on D24 melanoma cells (Figure 1D) but showed similar cytotoxicity in MM418-C1 melanoma cells (Figure 1E).

Furthermore, all three extracts showed double the level of cytotoxicity to the melanoma cells than HaCaT cells (Figure 1D–F). The higher cytotoxicity of the *M. cochinchinensis* seed extracts on both melanoma cell lines compared to that of HaCaT cells suggests that these extracts contain potential compounds specific to cancer cells.

### 2.2. Morphological Cellular Changes

**Figure 2:**
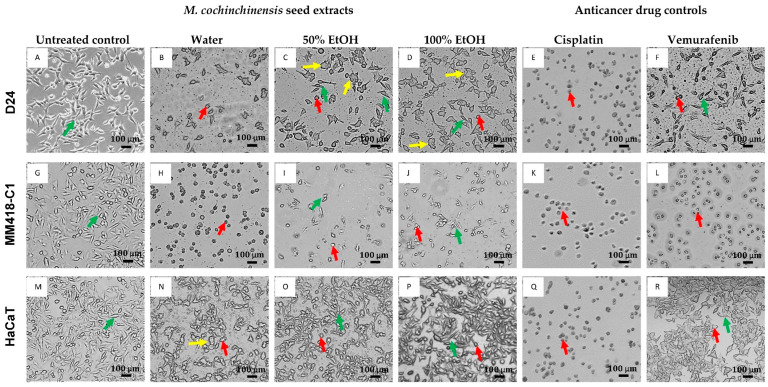
Effect of 48 h exposure to 100 µg/mL *M. cochinchinensis* seed extracts on melanoma (D24 and MM418-C1) and HaCaT cells compared to cisplatin and vemurafenib treatment. Green arrows indicate normal cells, red arrows indicate cell shrinkage, yellow arrows indicate cell swelling (Scale bar = 100 µm). (**A**): Untreated D24 cells (control), (**B**): D24 cells treated with water extract, (**C**): D24 cells treated with 50% EtOH extract, (D): D24 cells treated with 100% EtOH extract, (**E**): D24 cells treated with cisplatin, (**F**): D24 cells treated with vemurafenib, (**G**): Untreated MM418-C1 cells (control), (**H**): MM418-C1 cells treated with water extract, (**I**): MM418-C1 cells treated with 50% EtOH extract, (**J**): MM418-C1 cells treated with 100% EtOH extract, (**K**): MM418-C1 cells treated with cisplatin, (**L**): MM418-C1 cells treated with vemurafenib, (**M**): Untreated HaCaT cells (control), (**N**): HaCaT cells treated with water extract, (**O**): HaCaT cells treated with 50% EtOH extract, (**P**): HaCaT cells treated with 100% EtOH extract, (**Q**): HaCaT cells treated with cisplatin, (**R**): HaCaT cells treated with vemurafenib.

**Figure 3:**
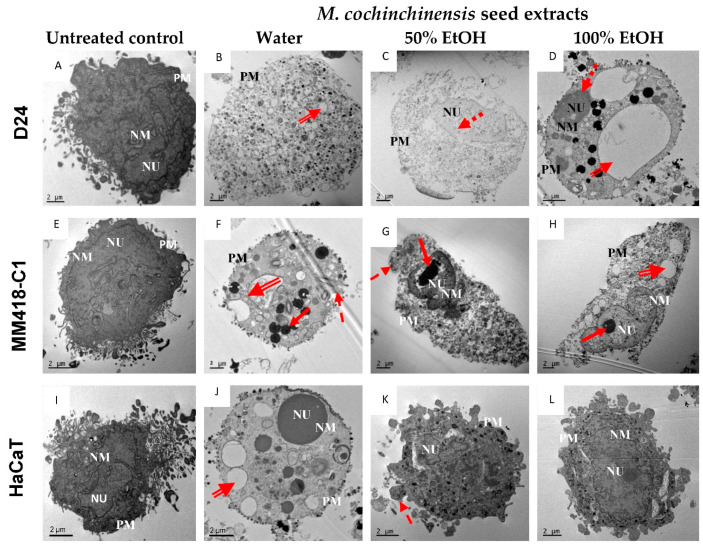
Effect of *M. cochinchinensis* seed extracts on the morphology of melanoma (D24 and MM418-C1) and HaCaT cells. The cells were treated with 100 µg/mL of the seed extracts for 48 h and examined under electron microscopy. Solid-line arrow: chromatin condensation, dotted-line arrow: margination of chromatin, hatched-line arrow: cell membrane blebbing, double-line arrow: vacuolisation, NU: nucleus, NM: nuclear membrane, PM: plasma membrane. (**A**): Untreated D24 cells (control), (**B**): D24 cells treated with water extract, (**C**): D24 cells treated with 50% EtOH extract, (**D**): D24 cells treated with 100% EtOH extract, (**E**): Untreated MM418-C1 cells (control), (**F**): MM418-C1 cells treated with water extract, (**G**): MM418-C1 cells treated with 50% EtOH extract, (**H**): MM418-C1 cells treated with 100% EtOH extract, (**I**): Untreated HaCaT cells (control), (**J**): HaCaT cells treated with water extract, (**K**): HaCaT cells treated with 50% EtOH extract, (**L**): HaCaT cells treated with 100% EtOH extract.

Changes in the morphology of the three cell lines were observed under both phase contrast (Figure 2) and transmission electron microscopy (Figure 3). The cells were treated with either 100 μg/mL of seed extracts or drug controls for 48 h and observed under phase contrast microscopy. The untreated D24 and MM418-C1 melanoma cells as well as the HaCaT cells displayed normal cellular morphology (Figure 2A,G,M). Following treatment with the water extract there were significant changes to the morphology of the MM418-C1 and D24 melanoma cells, as evidenced by cell shrinkage and rounding in shape (Figure 2B,H). These morphological changes were similar to that induced by cisplatin (Figure 2E,K), which reflected the results of the cell viability assay. Both the 50% and 100% ethanol seed extracts also induced morphological changes in the D24 (Figure 2C,D) and MM418-C1 melanoma cells (Figure 2I,J). Though not as pronounced as that seen for the water extract, cell shrinkage was predominant in MM418-C1 cells, and cell swelling was observed in D24 cells.

While cisplatin induced similar morphological changes in all three cell lines (Figure 2E,K,Q), the seed extracts and vemurafenib displayed selective effects with greater cytotoxicity seen in the melanoma cells than in the HaCaT cells. When these cells were treated with the water extract, there was a significant loss of cell density and shrinkage in both MM418-C1 and D24 cells (Figure 2B,H) which was not seen in the HaCaT cells (Figure 2N).

The effects of 100 µg/mL *M. cochinchinensis* seed extracts on the morphology of the cells treated for 48 h were observed under electron microscopy (Figure 3). The untreated cells each possessed a large nucleus containing distinct nucleoli and did not have any peripheral heterochromatin (Figure 3A,E,I). Both the water and ethanol seed extracts induced clear morphological changes in the melanoma cells, with most changes seen in water extract treatments (Figure 3B,F). When the D24 melanoma cells were treated with the water extract, the cells were swollen and the nuclear membrane and intracellular organelles had disintegrated along with the formation of a large number of vacuoles (Figure 3B), which were indicative of necrosis [29]. When the same cells were treated with the ethanol extracts, the nuclear membrane was still intact, chromatin margination can be observed (Figure 3C,D). While these characteristics indicate apoptosis, vacuolisation and disintegration of cell components in cytoplasm were also observed (Figure 3C,D) which is indicative of necrosis [29]. When the MM418-C1 melanoma cells were treated with water seed extracts, the disintegration of intracellular components and lysis of the nuclear membrane highlight necrosis (Figure 3F). The presence of a highly condensed nuclei, ruptured cellular organelles along with an intact plasma membrane, and decreased cell volume indicate late stage apoptosis or secondary necrosis (Figure 3F) [30]. When these cells were treated with the ethanolic seed extracts, ruptured cytoplasmic organelles in conjunction with an intact nuclear membrane, and condensed chromatin were observed suggesting early apoptotic changes (Figure 3G,H) [29]. These results suggest that both *M. cochinchinensis* water and ethanolic extracts contain compounds that can induce apoptosis and necrosis in these melanoma cells. As the dose used was 100 µg/mL which was twice that of the IC50 for the water extract, this may have triggered these cells to undergo necrosis.

The seed extracts had a lesser impact on the morphology of HaCaT cells (Figure 3J, K,L) compared to that observed in the melanoma cells. When HaCaT cells were treated with the 100% ethanol extract, they displayed large nucleoli within the nucleus but no peripheral heterochromatin (Figure 3L). On the other hand, treatment with the water extract resulted in a swollen cell containing large vacuoles and a condensed cytoplasm (Figure 3J). These morphological observations highlight the cytotoxic selectivity of the *M. cochinchinensis* seed extracts which was more cytotoxic toward melanoma cells than keratinocyte-derived cells.

### 2.3. Upregulation of TNFR1 Expression by M. cochinchinensis Seed Extracts Induced Melanoma Cell Death through the Suppression of the Nuclear Translocation of NF-kB

**Figure 4:**
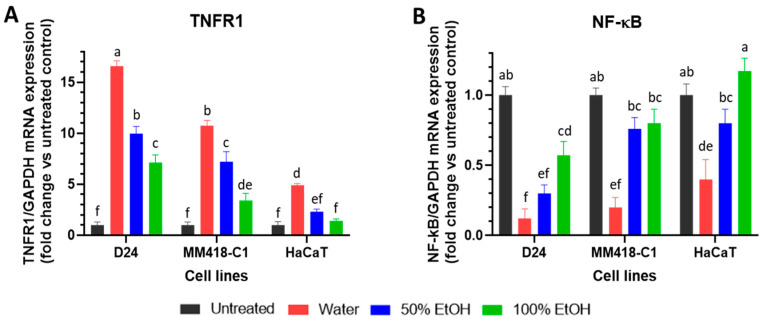
Effect of *M. cochinchinensis* seed extracts on the expression of. TNFR1 (**A**) and NF-kB (**B**) mRNA in D24 and MM418-C1 melanoma and HaCaT cells. The cells were treated with 100 µg/mL of the different seed extracts for 48 h prior to RNA collection. Results were presented as the fold change compared to untreated controls ± SEM (*n* = 3), different letters indicated statistical significance at *p* ≤ 0.05 between different treatments (Analysis by One-way ANOVA with Turkey’s LSD test).

Tumour necrosis factor alpha (TNF-α) can bind to two receptors-tumour necrosis factor receptor 1 (TNFR1) and tumour necrosis factor receptor 2 (TNFR2). On binding to TNFR1, TNF-α can trigger the extrinsic apoptotic pathway in the cell [31]. The *M. cochinchinensis* seed extracts upregulated TNFR1 mRNA expression in all 3 cell lines (Figure 4A). The greatest stimulation was seen with the water extract, where a 16-fold increase of TNFR1 mRNA was observed in D24 cells. A lesser increase was observed in the MM418-C1 and HaCaT cells (11- and 5-fold, respectively). The 50% and 100% ethanol seed extracts did not elicit the same stimulatory effect as did the water extracts in the cell lines that were tested.

The upregulation of TNFR1 can trigger both cell survival and apoptosis [32]. If the upregulation of TNFR1 is associated with the activation of NF-kB, it promotes cell survival and tumour growth [11]. If it does not cause an activation of NF-kB, then the extrinsic apoptotic pathway is initiated [32,33]. The seed extracts downregulated NF-kB expression in both melanoma cell lines (Figure 4B). In D24 cells, NF-kB mRNA levels fell 9.3-fold following treatment with the water seed extract, which was greater than that elicited by the 50% and 100% ethanol extracts (3.4- and 1.8-fold, respectively). In MM418-C1 cells, the water extract reduced NF-kB mRNA levels by 4.9-fold; however, both ethanol extracts had minimal effects (Figure 4B). In HaCaT cells, only the water extract downregulated NF-kB expression which was much less than that observed in both melanoma cells (Figure 4B).

### 2.4. M. cochinchinensis Seed Extracts Supressed Expression of MAPK Genes in the Melanoma Cells

**Figure 5:**
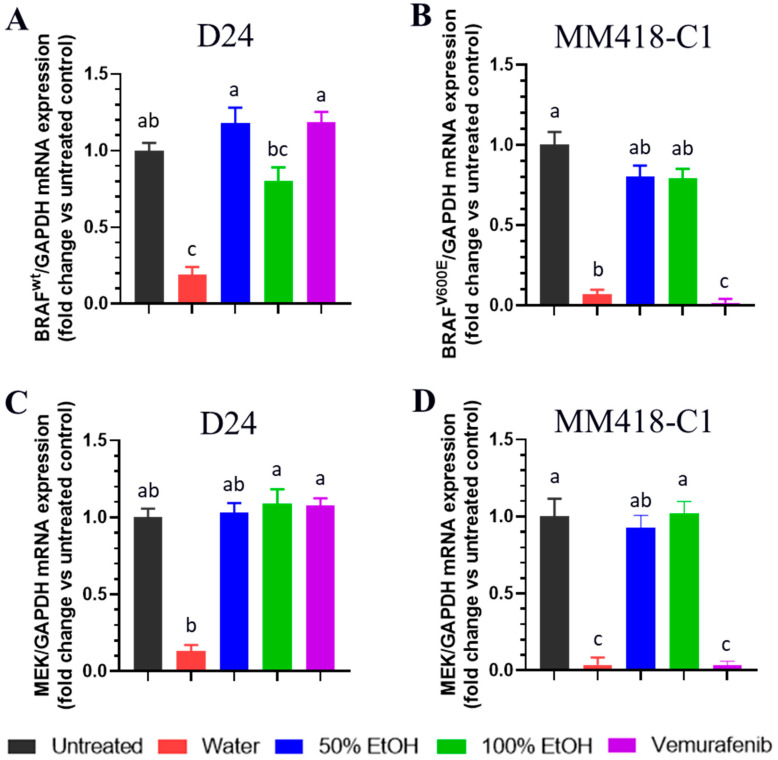
Effect of *M. cochinchinensis* seed extracts on the expression of BRAF*^WT^* gene in D24 (**A**), BRAF*^V600E^* gene in MM418-C1 (**B**), and MEK1 genes in D24 (**C**) and MM418-C1 (**D**) melanoma cells. The cells were treated with 100 µg/mL of the different seed extracts or vemurafenib for 48 h prior to RNA collection. Results were presented as the fold change compared to untreated controls ± SE, different letters indicated statistical significance at *p* ≤ 0.05 between different treatments (Analysis by One-way ANOVA with Turkey’s LSD test).

The V600E mutation is the most frequent BRAF mutation and is observed in ~90% of melanomas that carry this mutation [2]. The effect of the seed extracts and vemurafenib on the expression of BRAF*^WT^* and BRAF*^V600E^* genes in the melanoma cells were evaluated to detect BRAF mutation specific genomic changes. The water seed extract downregulated the expression of both BRAF*^WT^* (D24 cells) and BRAF*^V600E^* (MM418-C1 cells) by 5.1- and 14.9-fold, respectively (Figure 5A,B). Both ethanolic seed extracts did not have an effect on the expression of these genes. Vemurafenib downregulated BRAF*^V600E^* expression in the MM418-C1 cells by 72.4-fold (Figure 5B), which was 4.7-fold greater than that of the water seed extract. As expected, vemurafenib did not downregulate the expression of BRAF*^WT^* in the D24 melanoma cells.

The oncogenic BRAF*^V600^*^E^ mutation triggers the downstream members of the MAPK signalling pathway such as MEK1/2 and ERK1/2 which activates melanoma cell survival and proliferation [2,5]. MEK1 mRNA levels were significantly reduced in both MM418-C1 and D24 cells (18.8- and 7.8-fold, respectively) following treatment with the water seed extracts. Vemurafenib only downregulated MEK1 gene expression in MM418-C1 cells (Figure 5C,D). Similar to that seen for BRAF regulation, the ethanol extracts had no effect on MEK1 expression in both melanoma cell lines.

**Figure 6:**
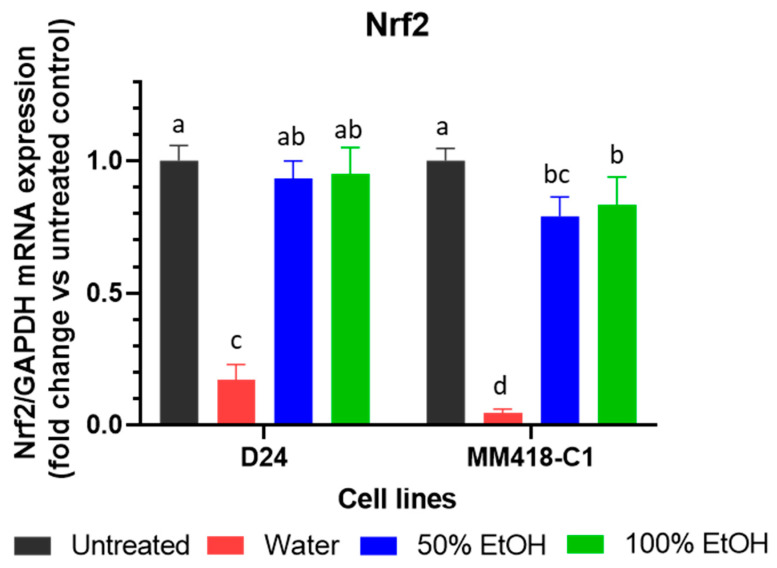
Effect of *M. cochinchinensis* seed extracts on the expression of Nrf2 mRNA in D24 and MM418-C1 melanoma cells. The cells were treated with 100 µg/mL of the different seed extracts for 48 h prior to RNA collection. Results were presented as the fold change compared to untreated controls ± SE, different letters indicated statistical significance at *p* ≤ 0.05 between different treatments (Analysis by One-way ANOVA with Turkey’s LSD test).

While BRAF and MEK inhibitors have revolutionized the treatment of melanoma patients [5] resistance frequently occurs after several months of treatment [2,5]. One of the main mechanisms triggering BRAF/MEK inhibitor-resistance in melanomas is activation of the Nrf2-pathway. This pathway enhances melanoma cells to overcome oxidative stress caused by BRAF/MEK inhibitors [34]. The expression of Nrf2 on both D24 and MM418-C1 melanoma cells after treatment with the seed extracts were examined (Figure 6). Only the water seed extracts significantly reduced Nrf2 expression in the MM418-C1 and D24 cells (11.6- and 5.9-fold, respectively). The ethanol seed extracts had no significant effects on the expression of Nrf2 in the MM418-C1 cells (Figure 6).

### 2.5. Proteins and Peptides in M. cochinchinensis Seed Extracts

**Figure 7:**
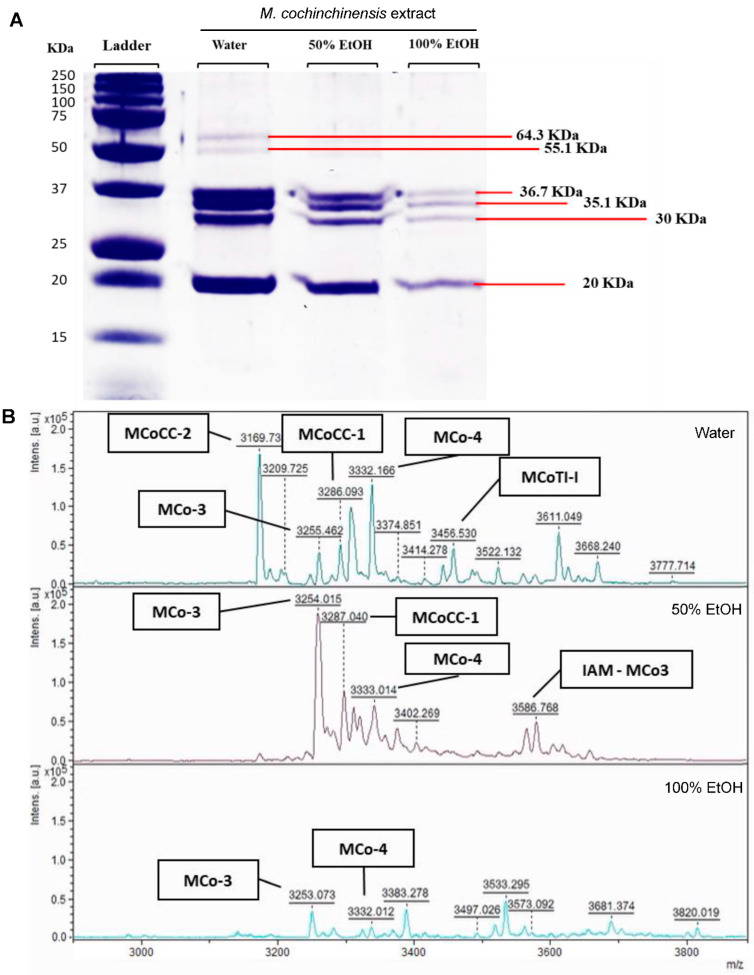
Protein (**A**) and peptide (**B**) profiles of *M. cochinchinensis* seed extracts.

The protein profile of the water and ethanol (50 and 100%) seed extracts were detected using SDS-PAGE assays. Four prominent proteins (20.0, 30, 35.1 and 36.7 kDa) were detected that were similar for all solvents (Figure 7A). In addition, two proteins (55.1 and 64.3 kDa) were detected only in the water extract. The concentration of the 4 proteins found in water extract was between 3.1–5.6-fold higher than that of the 100% ethanol extract. This difference suggests that these proteins are water soluble. The most prominent protein had a MW of 20 kDa, which was 18.9, 12.56 and 3.36.7 ng/mg in the water, 50% and 100% ethanol extracts, respectively (Table 1).

Mass spectra profiles of compounds between 1000 and 20,000 Da ranges were detected by MALDI-TOF. However, while there were no spectra peaks between 4000 and 20,000 Da, a number of peaks were recorded in the range between 3000 and 4000 Da. These peptides (3000 to 4000 Da) were detected for all solvents with the highest number of peptides and intensity observed in the water seed extract (12 peaks) (Figure 7B). Some 6 peptides unique to the water *M. cochinchinensis* seed extract were matched to known peptides including MCoCC-2, MCoCC-1, MCo-3, MCo-4, MCoTI-I and IAM-MCo3 based on published data [35]. MCoCC-2 was the most dominant peptide observed in the water extract but was not detected in either ethanol extracts (Figure 7B). The MCo-3 and MCo-4 peptides were observed in both the water and ethanol extracts. MCo-3 peptide was the dominant peptide observed in the 50% ethanol extract, which was ~3000 a.u. higher than that observed in the water and 100% ethanol extract (Figure 7B). These results confirm the presence of various peptides with molecular weights ranging between 3000–4000 Da in the *M. cochinchinensis* seed extracts. As these peptides have varying solubilities, the extraction solvent was an important factor that affected the efficiency of extraction and the diversity of peptides isolated.

### 2.6. Determination of Potential Oncogene Proteins from M. cochinchinensis Seed

The bioinformatic search of UniProt returned 33 proteins and peptides evidenced to be found in *M. cochinchinensis*. All identified peptides from MALDI-TOF mass spectrum were found in this search including MCoCC-2, MCoCC-1, MCo-3, MCo-4, MCoTI-I and IAM-MCo3 peptides. The search also revealed three proteins with molecular weights in the range of the proteins, i.e., from 20 to 37 kDa. This range was chosen as post-translational modifications could readily add or reduce the weight of these proteins. The three proteins were ribulose biphosphate carboxylase (uniprot code: D3W4J0, 26.2 kDa), two inhibitor peptide topologies 1 (TIPTOP 1) (uniprot code: J3RCD6, 29.3 kDa), and two inhibitor peptide topologies 2 (TIPTOP 2) uniprot code: J3R2I9, 34.4 kDa). Furthermore, literature also suggests a fourth protein Cochinin B [27] (28 kDa), however the full sequence of this is not available thus could not be analysed further.

In order to identify their potential targets in the human melanoma cell, a blast search was conducted on each of the proteins. This search identified Prolow-density lipoprotein receptor (LRP1, uniprot code: Q07954) as the closest human homolog for TIPTOP1 and TIPTOP2 with 25% and 23% identity, respectively. This receptor is involved in the endocytosis and phagocytosis of apoptotic cells. In the context of melanoma, LRP1 has been shown to regulate the metastatic behaviour of melanoma [36]. Its knockdown enhanced both the chemosensitivity [37] as well as reduced growth of these melanoma cells. For Ribulose biphosphate carboxylate, the closest human homolog was Tumour necrosis factor receptor superfamily member 12A (FN14) (uniprot code: I3L0S4) which had an amino acid identity of 41.2%. This protein has an established role in melanoma [38] with signalling confirmed to regulate oncogenic activity though MAPK, AKT and NF-kB pathways [39]. Further experimental work is needed to confirm the identity of these proteins.

### 2.7. Correlations between Cytotoxicity, Extraction Solvent, Gene Expression and Phytochemicals

**Figure 8:**
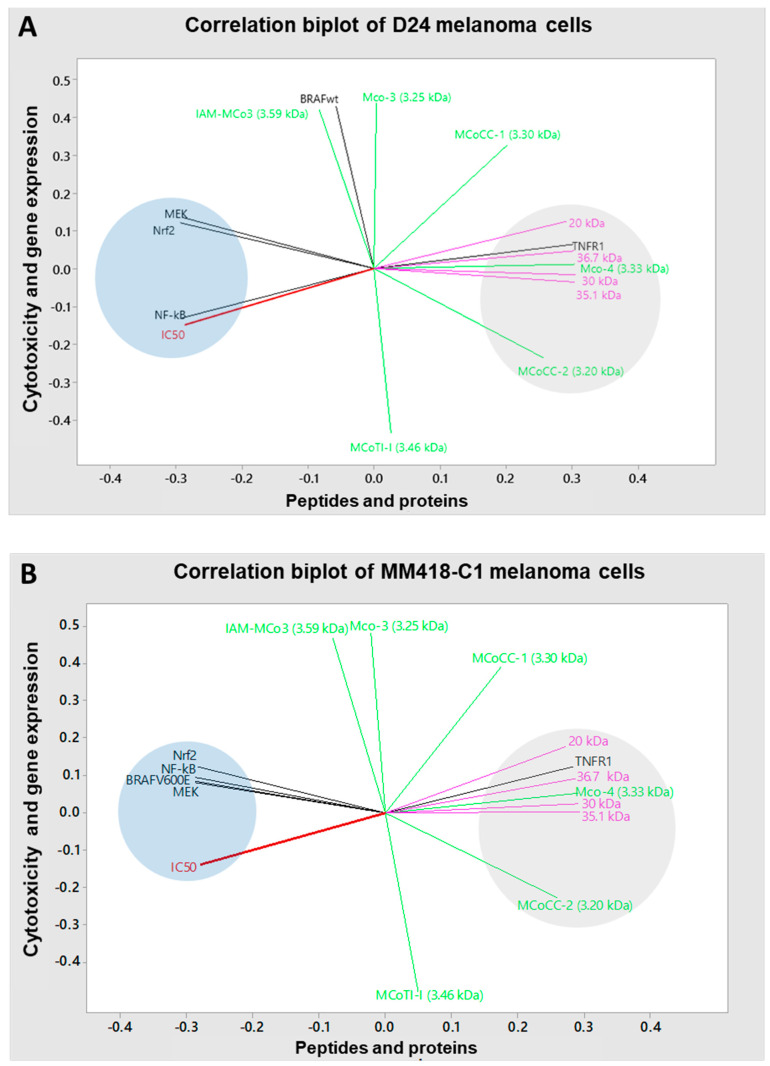
Correlation biplot of cytotoxicity (IC50) and gene expression with *M. cochinchinensis* seed proteins and peptides in D24 (**A**) and MM418-C1 melanoma cells (**B**) constructed using the principal component analysis method (PCA) with Minitab statistical software (Version 18). Red = IC50, black = gene expressions, purple = proteins and green = peptides.

The principal component analysis (PCA) method was used to analyse the correlations between the peptides/proteins contained in the seed extracts and IC50 values/mRNA expression levels of genes in D24 and MM418-C1 melanoma cell lines. In the D24 melanoma cells, cytotoxicity was strongly correlated with NF-kB (0.99, *p* < 0.001), MEK1 (0.81, *p* < 0.001) and Nrf2 (r = 0.79, *p* < 0.001) expression, indicated by the clustered grouping in the left quadrat (Figure 8A). These correlations suggested that the downregulation of NF-kB, MEK1 and Nrf2 genes were associated with decreased IC50 values. Additionally, this cluster showed close negative correlations with the second cluster of the PCA in the right quadrat (Figure 8A), which comprised seed proteins (20 kDa, 30 kDa, 35.1 kDa and 36.7 kDa, and peptides (MCo-4 and MCoCC-2). These negative correlations indicated that increased levels of these proteins and peptides were associated with reduced IC50 values.

In the MM418-C1 melanoma cells, cytotoxicity had a strong positive correlation with mRNA expression of MEK1 (0.90, *p* < 0.001), BRAF*^V600E^* (0.89, *p* < 0.001), NF-kB (r = 0.85, *p* < 0.001) and Nrf2 (r = 0.79, *p* < 0.001), as seen clustered together in the left quadrat (Figure 8B). These correlations indicated that cytotoxicity elicited by the seed extracts was accompanied with the downregulation of these genes. Furthermore, high levels of various proteins and peptides in the seed extracts was responsible for the reduced viability of the melanoma cells as the IC50 values displayed inversed correlations with the seed proteins (20 kDa, 30 kDa, 35.1 kDa and 36.7 kDa), and peptides (MCo-4 and MCoCC-2) in the right quadrat cluster (Figure 8B). In contrast, other peptides in the seed extract such as IAM-MCo-3, MCo-3 and MCoTI-I effects had a weaker correlation with the IC50 value, which might indicate less cytotoxic effects on the melanoma cells.

The upregulation of TNFR1 mRNA expression in both melanoma cell lines correlated closely to the protein levels in the seed extracts, especially to the 36.7 kDa (r = 0.99, *p* < 0.001) and 20 kDa (r = 0.97, *p* < 0.001) proteins, whilst there were weak correlations with IAM-MCo-3, MCo-3 and MCoTI-I peptides (Figure 8A,B). This indicated that the upregulation of TNFR1 was associated with proteins in the seed extracts rather than peptides. In contrast, the downregulation of NF-kB, MEK1 and Nrf2 expression in both D24 and MM418-C1 cells were inversely correlated with MCo-4 and MCoCC-2 proteins and peptides with r values from -0.85 to -0.98 (Figure 8A,B). Interestingly, the MCoCC-2 peptide displayed a close inverse correlation with the reduced expression of BRAF*^V600E^* (r = −0.98, *p* < 0.001) and MEK1 (r = −0.96, *p* < 0.001), while the proteins displayed lower negative correlations to these genes (Figure 8B). These results suggest that high levels of proteins as well as the MCo-4 and MCoCC-2 peptides in the seed extracts are likely responsible for the cytotoxic effects observed on the melanoma cells, most likely through triggering the TNFR1/NF-kB and BRAF/MEK1/Nrf2 induced cell death pathways.

## 3. Discussion

*M. cochinchinensis* seeds were effective at reducing melanoma cell viability and the best extraction solvent was water. These aqueous extracts displayed for the first time, comparable IC50 values to that of vemurafenib controls on MM418-C1 cells, and cisplatin controls on D24 melanoma cells. Cisplatin is used in the treatment of various malignant cancers such as melanoma, ovarian, breast, leukemia and lymphomas [40], while vemurafenib is used to treat melanomas carrying the BRAF*^V600E^* mutation [2]. Since our water extracts have similar bioactivity as these generic and specific drugs, this offers an exciting possibility as a natural herbal alternative to current cancer treatments and provides the preliminary evidence for further clinical studies to determine optimal dosages and safety pharmacokinetics.

The cytotoxic effect of the water seed extract on the melanoma cells was likely due to specific proteins and peptides unique to *M. cochinchinensis*. In this study, the low IC50 of the water seed extracts on both melanoma cells had strong inverse correlations with the MCo4 peptide and 20 kDa, 30 kDa, 35.1 kDa and 36.7 kDa proteins. The 20 kDa and 36.7 kDa proteins have not been detected in other studies on *M. cochinchinensis* seed extracts [17,27,41]. The other proteins have demonstrated bioactivity in other studies and were likely the responsible anticancer agents; where a 35 kDa protein exerted cytotoxic effects on HepG2 hepatoma and 26–20 colon cancer cells [17]; and 30 kDa protein [26] on cervical epithelial carcinoma.

This is the first bioinformatic analysis of the proteins that was suggested to be ribulose biphosphate carboxylate and inhibitor peptide topologies (TIPTOP 1 and 2). All three proteins have human homologs, LRP1 and FN14, that have established roles in melanoma [36,37,38,39]. This suggests these *M. cochinchinensis* proteins could be competitive inhibitors of their human counterparts.

It is possible that the native mix of proteins and peptides found in the crude seed extracts exert a synergistic effect, and when isolated as purified compounds may not be as effective and was the motivation for our studies. Others have found that isolated and purified MCoCC-1 and MCoCC-2 peptides from *M. cochinchinensis* seeds were less effective against MM96L melanoma cells than the equivalent concentration of the crude seed extract mixture [25]. Based on our limited proteomic evaluations, there was a range of proteins and peptides in the native seed mixture that warrant further characterisation for improved bioactivity, possibly on other cancers.

The cytotoxicity of the seed extracts was dose-dependent, with higher concentrations exerting higher cell death. The water extracts were not specific to genetic mutations and were effective on both BRAF*^WT^* and BRAF*^V600E^* mutations. Since BRAF MM418-C1 cells possess the oncogenic BRAF*^V600E^* mutation while D24 and HaCaT cells do not [42], this could also suggest that a single compound has non-specific action or that there are multiple effective compounds. Therefore, the water extract could potentially be a universal treatment for various types of cancers, not just to melanoma. Since the water extract contains a wide range of proteins and peptides, these are likely to target different cell death pathways as shown in the PCA. Furthermore, the seed extracts were more cytotoxic against melanoma cells than the keratinocyte-derived HaCaT cells and highlights the potential use of *M. cochinchinensis* seeds to specifically target melanoma.

Although ethanol seed extracts exerted lower cytotoxic effects on the cancer cells than did the water extract, it was still effective. These ethanol extracts from *M. cochinchinensis* seeds also displayed cytotoxic effects on MKN-28 and SGC7901 gastric cancer, A549 lung cancer, and cMDA-MB-301 breast cancer cells with IC50 values of 200, 380, 390 and 310 µg/mL, respectively [18,19,20], which were 2–4 times higher than that elicited by the ethanol seed extracts on our D24 and MM418-C1 melanoma cells.

Treatment with the seed extracts altered the expression of TNFR1 and NF-kB mRNA in the melanoma cells, which in turn triggered both apoptosis and necrosis. TNF-α is a pro-inflammatory cytokine that balances homeostasis by regulating the production of cytokines, cell survival, and cell death by apoptosis and/or necrosis [33]. It can bind to one of two receptors, TNFR1 or TNFR2 [33]. If TNF-α binds to TNFR1, it can promote apoptosis, however when it binds to TNFR2, it is anti-apoptotic [43]. Increased membrane levels of TNFR1 associated with TNFα-TNFR1-induced apoptosis in melanoma cells [44].

TNF-α can trigger the cell death pathway only when it does not activate NF-kB. In this study, increased cell death was observed after exposure to seed extracts, which correlated with increased TNFR1 and reduced NF-kB mRNA expression levels. Furthermore, the upregulation of TNFR1 and downregulation of NF-kB and Nrf2 were significantly greater when these cells were treated with the water extract rather than with the ethanol extracts. This result was consistent with the reduction of cell viability and morphological changes observed in the D24 and MM418-C1 melanoma cells. The water extract had a significantly lower IC50 value and elicited greater morphological damage than did the ethanol extracts. It was clear that these cells had undergone apoptosis as well as necrosis. The upregulation of TNFR1 can form complex IIa (composed of TNF-α/TNFR1, TRADD, FAS associated death domain (FADD) and pro-caspase-8), and complex IIb (composed of TNF-α/TNFR1 and RIPK1) trigger apoptosis via the caspase-8 pathway [32,33]. However, if the TNF-α/TNFR1 complex bind to the mixed lineage kinase domain-like protein (MLKL), it forms complex IIc, which promotes necrosis in the cell [33]. Since the results from the morphological studies display both apoptotic and necrotic characteristics, the activation of TNFR1 and suppression of NF-kB would most likely trigger the formation of complex IIa and IIb which induces apoptosis, as well as complex IIc which triggers the necrosis in these cells as well. Of note, one of our identified proteins, ribulose biphosphate carboxylate, has a human homolog, FN14, which has a known role in the TNFR1/NF-kB pathway [37].

The BRAF gene encodes for the BRAF serine/threonine kinase that is part of the RAS-RAF-MEK-MAPK signalling pathway [2]. Mutations of BRAF are the most prevalent genetic alteration in human melanoma, with ~50% of tumours expressing the BRAF*^V600E^* oncoprotein [7]. Inhibition of the BRAF/MEK pathway leads to apoptosis, and the suppression of cell proliferation and metastasis in melanoma cells [7]. Given the significance of BRAF activation in melanoma cells, the down-regulation of both BRAF*^WT^* (D24 melanoma cells) and BRAF*^V600E^* (MM418-C1) in the water seed extract treated cells assumes importance. The water seed extract exerted a similar effect on inhibiting BRAF mRNA expression to that of the specific BRAF*^V600E^* inhibitor vemurafenib in MM418-C1 cells. Of interest was that the water extract also inhibited BRAF mRNA expression in the D24 BRAF*^WT^* cells, this differed to vemurafenib which enhanced expression. A similar observation was observed when comparing the water extract and vemurafenib on MEK1 expression in both melanoma cell lines. These results suggest that the water extract possesses multiple compounds that are BRAF*^WT^*, BRAF*^V600E^* and MEK inhibitors. However, on normal epithelial skin cells such as HaCaT cells, that do not contain BRAF mutations, the water extract had a lower cytotoxic effect than on MM418-C1 cells which possessed the BRAF mutation.

The transcription factor Nrf2 is the major mediator of oxidative stress responses and is closely connected to therapy resistance in tumours harbouring activating mutations in this pathway [45]. High Nrf2 expression correlates with low survival rates in melanoma patients by triggering infinite cell growth and proliferation, reduced apoptotic death, enhanced chemoresistance and radioresistance, and induced both angiogenesis and metastasis in these tumours [34,45,46]. Jessen et al. (2020) reported that BRAF*^V600E^* melanoma cells have high levels of Nrf2 gene expression, and blocking it resulted in the suppression of the growth of these cancer cells [34]. Increased Nrf2 expression via enhanced MAPK activity is a result of BRAF mutations [34]. It is likely that the water seed extract downregulated Nrf2 levels as a result of its inhibition of BRAF and MEK in the MAPK pathway.

Changes in TNFR1 mRNA levels closely correlated with that of the 20, 30, 35.1 and 36.7 kDa proteins whilst that of BRAF*^WT^*, BRAF*^V600E^* and MEK1 mRNA had a strong negative correlation to the MCoCC-2 peptide. The proteins found in the seed extracts might be too large to be taken up by the cell [47], but may interact with cell surface receptors. For example, the human homolog of ribulose biphosphate carboxylate, Fn14, interacts with TWEAK which regulates TNFR1 and TNFR2 [48]. Ribulose biphosphate carboxylate may mimic this interaction and act as a competitive inhibitor of TNFR regulation. In contrast, the smaller sized MCoCC-1 and MCoCC-2 peptides (<3.3 kDa) could permeate through the cell membrane and target intracellular signalling pathways such as BRAF, MEK and Nrf2 [47]. MCoCC-2 is a cysteine knot peptide and has no sequence homology with any previously characterised peptides and are unique to the *M. cochinchinensis* seed [35]. This peptide has been shown to be resistant to thermal, chemical, or proteolytic degradation 35, and could be useful as a peptide-based drug. The strong negative correlation of MCoCC-2 levels and that of MAPK (BRAF*^V600E^* and MEK1) gene expression suggested that it could be useful as a potential inhibitor of this pathway in melanoma cells. However, further studies are needed to validate and confirm if this is the case.

## 4. Materials and Methods

### 4.1. Materials

RPMI-1640 media, heat-inactivated foetal bovine serum (FBS), streptomycin, penicillin, cisplatin and ethanol were obtained from Thermo Fisher Scientific (Melbourne, Australia). Cell Counting Kit-8 (CCK-8) and TRIzol regent were purchased from Sigma-Aldrich (Sydney, Australia). TNFR1, NF-kB and Nrf2 primers were purchased from Bioneer Pacific (Melbourne, Australia), SensiFAST SYBR No-ROX qPCR and superscript III reverse transcriptase kits were purchased from Bioline (Sydney, Australia).

### 4.2. Preparation of Extracts

*M. cochinchinensis* seeds collected from cultivated plants in Lam Dong Province Vietnam in 2013. Identification was confirmed by indigenous locals and molecular studies, and voucher specimens deposited at the national Herbarium of Victoria (Accession MEL2472087). Whole plants were frozen and transported to Australia in darkness. The seeds were separated from frozen ripe fruits collected from Vietnam. Firstly, the seeds were decoated and crushed into a powder using a mortar and pestle. Then, to each 2 g of seed powder, 100 mL of solvent (water, 50% or 100% ethanol) was added and covered with aluminium foil to protect from sunlight. The mixtures were ultra-sonicated for 15 min and shaken for 24 h using an orbital mixer before being filtered through 0.45 µm Whatman^®^ qualitative filter paper (Grade 1). The water mixture was freeze dried in an Alpha 1-2 LDplus Entry Freeze Dryer. The organic solvent in the 50% and 100% ethanol mixtures were evaporated using RVC 2-33 CDplus speed vacuum evaporator, before being freeze dried to obtain a dry powdered extract. The crude extracts were stored at -80°C until further use.

The water extract was dissolved in milliQ water, while both ethanol extracts were dissolved in 0.09% (v/v) DMSO at a concentration of 11 mg/mL. The extracts were incubated in a water bath sonicator for 10 min prior to being added to 60% confluent cell cultures grown in 96 well plates to give a final concentration of 0, 15.625, 31.25, 62.5, 125, 250, 500 and 1000 μg/mL.

### 4.3. Cell Culture Initial Growth

Human melanoma MM418-C1 (1° melanoma BRAF*^V600E^* oncogene) and D24 (2° melanoma BRAF*^WT^*) cells, and human immortalised keratinocytes (HaCaT) were used. Cells were cultured in RPMI-1640 medium supplemented with 10% (v/v) FBS and 1% (v/v) penicillin and streptomycin. The cells were incubated at 37 °C in a humidified 5% CO_2_ incubator and passaged every 3–4 days at 80–90% confluency.

### 4.4. Cytotoxicity Assays

Cytotoxicity was determined using the Cell Counting Kit-8 (CCK-8) as per the manufacturer’s instructions. Cells were seeded at 3 × 103 cells/well in 96 well plates containing RPMI-1640 media with 10% FBS and 1% (v/v) penicillin and streptomycin. Cells were allowed to adhere for 48 h at 37 °C in 5% CO_2_ before being serum-starved with 2% FBS for 12 h. After which they were treated with the seed extracts or anticancer drugs cisplatin or vemurafenib (0 to 1000 µg/mL) while the corresponding controls contained either water or 0.09% DMSO for 48 h. At the end of this period, CCK-8 was added to the cells for 2 h and the resultant absorbance was measured spectrophotometrically at 450 nm using a CLARIOstar^®^ High Performance Monochromator Multimode Microplate Reader. All tests and analyses were performed in triplicate. Viability was expressed as a percentage of the corresponding untreated controls which were assigned a value of 100%. The half-inhibition concentration (IC50) was determined using dose response-inhibition analysed by GraphPad Prism 9 software.

### 4.5. Morphological Changes of Cells Using Transmission Electron Micrography (TEM)

Preparation for observing cell morphology using TEM imaging was as previously described [49] with slight modifications. Cells were treated with 100 µg/mL seed extracts for 48 h. After which they were washed with RPMI-1640 media plus 10% FBS and was immersed in 2.5% (v/v) glutaraldehyde and 2% (v/v) paraformaldehyde in 0.1 M cacodylate buffer (pH 7.3) and left overnight at 4 °C. The cells were then trypsinised and centrifuged at 400× *g* for 5 min, before the pellets were fixed with 1% (v/v) OsO4 and 1.5% (v/v) potassium ferrocyanide for 1.5 h and washed twice with distilled water for 10 min. Dehydration was conducted in a series of ethanol steps for 15 min (50, 70, 90, 95%) followed by 100% (v/v) ethanol for 30 min and repeated, before being immersed twice in 100% (v/v) acetone for 30 min. Infiltration was carried out using a mixture of acetone and Spurr’s resin mix (1:1) on a shaker overnight at RT. The next day, new acetone: Spurr’s resin mix (1:1) was added to the cells and left for 2 h. Then, 100% of Spurr’s resin was added to the cells and placed under vacuum for 2 h. This was followed by exchanging to fresh resin and continuing the infiltration for 2 h. Finally, the cells were embedded and cured in resin at 70 °C for 24 h. Sectioning was done using an ultra-microtome (Leica Ultracut UCT) to produce thin <1 μm sections. The thin sections were washed with distilled water and dried on blotting paper, prior to being examined under a JEOL1010 transmission electron microscope equipped with a Gatan Orius SC600 CCD Camera.

### 4.6. Gene Expression of TNFR1, NF-kB, BRAF, MEK1 and Nrf2

The cells were seeded with 3 × 10^5^ cells/well in 6 well plates containing RPMI media plus 10% (v/v) FBS and 1% (v/v) penicillin and streptomycin. Cells were incubated at 37 °C in a humidified 5% CO_2_ incubator until the cultures reached ~75% confluency. The cultures were serum starved in 2% FBS RPMI media for 24 h, before being treated with 100 µg/mL of the seed extracts for 48 h. RNA was extracted using TRIzol™ Reagent (Life Technologies, Melbourne, Australia) as per the manufacturer’s protocol. RNA was quantified spectrophotometrically at 260 nm (Nanodrop ND1000), and its purity checked using the ratios of absorbance at 260/280 nm and 260/230 nm. cDNA was synthesized from 1 μg total RNA using SuperScript III reverse transcriptase (Thermo Fisher Scientific) with oligo (dT) primers according to the manufacturer’s instructions. Each transcript quantification was carried out in triplicate. Subsequently, 4 µL (10 ng/µL) cDNA was added to 10 µL SYBR mix, 0.8 µL of each (10 µM) forward and reverse primer, and 4.4 µL DEPC H_2_O. The qPCR was performed on a Qiagen Rotor-Gene Q (Qiagen, Germany) and analysed with inbuilt software (version 2.3.1.49). The thermal cycling conditions were as follows: initial activation at 95 °C for 2 min, 39 cycles of 95 °C for 5 s, 60 °C for 10 s, 72 °C for 15 s, and melting curve from 65 °C to 95 °C. Relative quantitative analysis of TNFR1, NF-kB, BRAF*^WT^*, BRAF*^V600E^* and Nrf2 mRNAs were performed using the 2^−ΔΔCq^ method which was normalized to glyceraldehyde-3-phosphate dehydrogenase (GAPDH) mRNA. The expression was calculated as *n*-fold induction of the gene of interest in treated cells relative to that of untreated control cells. The amplification efficiency (E) for each primer pair was determined using a cDNA dilution series (Table 2).

### 4.7. Protein and Peptide Profile Analysis

The protein and peptide profiles of the *M. cochinchinensis* seed extracts were investigated using SDS-PAGE electrophoresis and matrix-assisted laser desorption/ionization time-of-flight mass spectrometry (MALDI-TOF-MS).

### 4.8. SDS-PAGE Electrophoresis

Protein profiles of the seed extracts were detected using SDS-PAGE electrophoresis. The separating gel contained 16% (w/v) acrylamide while the stacking gel contained 5% (w/v) acrylamide. To each lane 10 µL seed extract or protein marker (Odyssey One-Color molecular marker) was mixed with 2.5 μL mercaptoethanol, and 7.5 μL of 0.002% bromophenol blue in 0.0625 mM Tris-HCl (pH 6.8), containing 10% glycerol and 2% SDS was added. The gel was run for 60 min at 100 V and 0.35 A using a Mini-PROTEAN Tetra Cell (Bio-Rad). At the end of the run, the gel was stained with 0.1% Coomassie Brilliant Blue R250 in 45% (v/v) methanol and 10% (v/v) glacial acetic acid before being destained in 10% (v/v) methanol. It was then washed with distilled water until clear and photographed with an Alpha Innotech Alphaimager HP MultiImage II. The molecular weights and concentrations of proteins of interest were calculated relative to the Odyssey One-Color molecular marker (Millennium Science, Sydney, Australia) using ImageLab software version 5.2 build 4 (Bio-Rad).

### 4.9. Putative Peptides Detected Using MALDI-TOF-MS

Putative detection of peptides in the seed extract using MALDI-TOF-MS was prepared as previous study [25]. Triplicate 1 µL of the seed extracts (1 mg/mL) were spotted onto a MALDI plate (AnchorChip). After drying, 1 µL of MALDI matrix HCCA was added to each sample spot. The HCCA solution consisted of 10 mg of α-cyano-4-hydroxycinnamic acid dissolved in 70% (v/v) acetonitrile (ACN) containing 0.1% (v/v) trifluoroacetic acid (TFA). The spots were air dried so that the samples could co-crystallize with the matrix. Mass spectra profiles of the seed extracts were obtained using an Autoflex Speed MALDI-TOF instrument (Bruker, Germany) equipped with a Nd:YAG laser operating at 355 nm, a laser frequency of 50 Hz with 500 laser shots and an acceleration voltage of 20 kV. Data were collected between mass range of 1000 to 20,000 Da. Data from the plate was collected and analysed using the Flex Control software.

### 4.10. Bioinformatic Analysis of Potential Oncogene Proteins

The UniProt database [50] was used to search for any published proteins and peptides from *M. cochinchinensis* fruit. Keywords such as ‘Gac fruit’, ‘Gac seed’, ‘Gac seed protein’, ‘Gac seed peptide’, ‘Spiny bitter cucumber’ and ‘*Momordica cochinchinensis*’ were all inserted into the search tool to cover a wide range of preferred names. Proteins identified around the approximate weight of the extracts were selected and Blast was utilised to determine any human homologues.

### 4.11. Statistical Analysis

The results were analysed with the one-way analysis of variance (ANOVA) using Minitab statistical software (version 18). For normally distributed data, the means were compared using the one-way analysis of variance (ANOVA) and Fisher’s post hoc test. Pearson’s correlation was used to test the correlation between cytotoxicity and eco-geographical parameters. Statistical values of *p*  <  0.05 were considered as significantly different. The inhibitory concentration (IC50) for cytotoxicity was derived from a nonlinear regression model (curve fit) based on a sigmoidal dose response curve (variable) and computed using GraphPad Prism version 6. The principal component analysis (PCA) was used to determine anticancer activity of the seed extract of *M. cochinchinensis* using Minitab statistical software (version 18).

## 5. Conclusions

Taken together, the findings from this study highlighted that *M. cochinchinensis* seeds extracted with water possessed cytotoxic activity against both D24 and MM418-C1 melanoma cell lines, but was less toxic to normal epidermal keratinocytes. The water extracts contained high levels of proteins and peptides likely due to their hydrophilic nature, and was more cytotoxic than the corresponding ethanol extracts for both melanoma cell lines. Water is the most suitable solvent for extracting these compounds giving an advantage for *M. cochinchinensis* seeds as a potential source of anticancer compounds that can be easily extracted with a green solvent [51]. Mechanisms of the antimelanoma activity were from induction of apoptotic and necrotic cell death pathways by triggering TNFR1 and supressing of NF-kB expression. The seed extracts also repressed MAPK pathway through inhibiting the expression of MEK and BRAF*^WT^* in D24 melanoma cells, and BRAF*^V600E^* in MM418-C1 cells. Proteins present in the seed extract were most likely responsible for altering TNFR1/NF-kB pathway activity, while the smaller sized peptides downregulated the BRAF/MEK pathway. This suggested that *M. cochinchinensis* seed extracts contains multiple anticancer compounds that have a synergistic effect on melanoma cells irrespective of their BRAF status. This is the first study to report on the molecular mechanism of action of *M. cochinchinensis* seed extracts on BRAF mutant melanoma cells. Since the water extract demonstrated equivalent cytotoxic effects as cisplatin and vemurafenib on melanoma cells, it is suitable for development as an anticancer alternative and complementary medicine. Further clinical trials on animals and humans will be required to determine the comprehensive mechanisms of actions, and evaluate the potential of this natural source of anticancer compounds.

## Figures and Tables

**Table 1:** Concentration of proteins in *M. cochinchinensis* seed extracts. Protein concentrations were calculated relative to the Odyssey One-Color molecular marker (Millennium Science, Sydney, Australia) and ImageLab software (Bio-Rad, Hercules, CA, USA). Results were presented as mean ± SEM (*n* = 3), values in each row with different letters indicated statistical significance at *p* ≤ 0.05 (Analysis by One-way ANOVA with Turkey’s LSD test).

Proteins (kDa)	Protein Concentration (ng/mg)		
	Water	50% EtOH	100% EtOH
20.0	18.9 ± 1.98 ^a^	12.7 ± 1.32 ^b^	3.4 ± 0.97 ^c^
30.0	7.9 ± 1.03 ^a^	4.8 ± 0.88 ^b^	1.2 ± 0.63 ^c^
35.1	8.7 ± 0.93 ^a^	4.5 ± 0.76 ^b^	1.8 ± 0.64 ^c^
36.7	6.5 ± 1.09 ^a^	3.8 ± 0.79 ^b^	1.3 ± 0.82 ^c^
55.1	1.7 ± 0.54 ^a^	0.00 ^b^	0.00 ^b^
64.3	2.1 ± 0.35 ^a^	0.00 ^b^	0.00 ^b^

**Table 2:** Primers used in this study and their amplification efficiencies.

Primer	Forward Sequence (5′-3′)	Reverse Sequence (5′-3′)	Amplification Efficiency (E) (%)
TNFR1	GGGCACCTTTACGGCTTCC	GGTTCTCCTTACAGCCACACA	99
NF-kB	ATAGAAGAGCAGCGTGGGGACT	GGATGACGTAAAGGGATAGGGC	98
Nrf2	AGTGGATCTGCCAACTACTC	CATCTACAAACGGGAATGTCTG	99
BRAF*^WT^*	GGCAGAGTGCCTCAAAAAGAA	AACCAGCCCGATTCAAGGA	99
BRAF*^V600E^*	CCGACCAGCAGATGAAGATCAT	TCAACATTTTCACTGCCACATCAC	99
MEK1	CAGAAGAAGCTGGAGGAGCTAG	CCATCGCTGTAGAACGCACCAT	97
GADPH	TCCACCACCCTGTTGCTGTA	ACCACAGTCCATGCCATCAC	99

## Data Availability

All data generated or analysed during this study are included in this published article and its additional flies. Gene expression data was deposited in Gene Expression Omnibus, reference number GSE2006541 (www.ncbi.nlm.nih.gov/geo/).
